# Dissemination of fusidic acid resistance among *Staphylococcus aureus* clinical isolates

**DOI:** 10.1186/s12866-015-0552-z

**Published:** 2015-10-13

**Authors:** Fangyou Yu, Yunling Liu, Chaohui Lu, Jinnan LV, Xiuqin Qi, Yu Ding, Dan Li, Xiaoying Huang, Longhua Hu, Liangxing Wang

**Affiliations:** Department of Laboratory Medicine, the First Affiliated Hospital of Wenzhou Medical University, Wenzhou, 325000 China; Department of Respiratory Medicine, the First Affiliated Hospital of Wenzhou Medical University, Wenzhou, 325000 China; Department of Laboratory Medicine, the Second Affiliated Hospital of Nanchang University, Nanchang, 336000 China

**Keywords:** *Staphylococcus aureus*, Fusidic acid, Resistance, Resistance determinants, Molecular characteristic

## Abstract

**Background:**

A significant trend towards increased fusidic acid (FA) resistance among *Staphylococcus aureus* with increased duration of use is of concern. The aim of the present study is to investigate the dissemination of fusidic acid resistance among *Staphylococcus aureus* clinical isolates.

**Methods:**

The susceptibility of *S. aureus* isolates to antimicrobial agents was determined by disc-diffusion method. The minimal inhibitory concertrations(MICs) of fusidic acid and vacomycin for fusidic acid-resisitant isolates were determined by ager dillution method. FA resistance determinants were determined by PCR and DNA sequencing. SCC*mec* typing, *spa* typing and multi-locus sequence typing were used for the determination of molecular characteristics for *S. aureus* isolates.

**Results:**

A total of 392 non-duplicate *S. aureus* isolates including 181 methicillin-resistant *S. aureus* (MRSA) isolates, which were isolated from the clinical specimens of patients at a Chinese tertiary hospital from January, 2012 to September, 2013, were collected for investigating FA resistance. Among 392 *S. aureus* clinical isolates tested, 56 (14.3 %) with FA MIC values ranging from 2 μg/ml to ≥128 μg/ml were resistant to FA. The proportions of FA resistance among MRSA and MSSA isolates were 27.1 % (49/181) and 3.3 % (7/211). There was a trend of rapidly increased FA resistance among *S. aureus* and MRSA isolates from 5.2 % and 8.9 % in 2012 to 24.9 % and 45.1 % in 2013. Acquired FA resistance gene, *fusB*, was present in 73.2 % (41/56) of FA-resistant *S. aureus* isolates. *fusC* and *fusA* mutation were not found in any of tested isolates. A total of 9 sequence types (STs) and 12 *spa* types were identified among the 56 FA-resistant *S. aureus* isolates. ST5 accounting for 66.1 % (37/56) was the most prevalent ST. The majority (92.9 %, 52/56) of the isolates tested belonged to clonal complex 5(CC5). *t*2460 was the most prevalent *spa* type, accounting for 67.9 % (38/56) . ST5-MRSA- II-*t*2460 was predominant clone, accounting for 75.5 % (37/49) of FA-resistant MRSA isolates and 66.1 % (37/56) of FA-resistant *S. aureus* isolates. Five of 7 FA-resistant MSSA isolates belonged to ST630-MSSA.

**Conclusion:**

Increased FA resistance among *S. aureus* isolates was found in China. *fusB* was predominant FA resistance determinant. The spread of CC5 clone, especially novel ST5-MRSA- II-*t*2460 clone with high-level resistance to FA, was responsible for the increase of FA resistance.

## Background

*Staphylococcus aureus*, especially methicillin-resistant *S. aureus* (MRSA), is an important and often encountered human pathogen responsible for many infectious diseases from mild superficial skin infection to life-threatening diseases, including skin and soft tissue infections (SSTIs), foreign-body infections, pneumonia, septic arthritis, endovascular infections, osteomyelitis, sepsis, and bloodstream infections (BSI) in both hospitals and community settings [1]. Since the first European isolate of MRSA was detected in 1960s, MRSA with resistance to all available penicillins and other β-lactam antimicrobial agents has become a leading cause of nosocomial infections in hospitals and other healthcare facilities. In addition to β-lactam, MRSA isolates are often resistant to erythromycin, clindamycin, aminoglycosides and quinolones. Since the mid-1990s, however, MRSA isolates are found to be responsible for severe infections in individuals lacking risk factors for exposure to the health care system [1, 2]. MRSA infections has become a global concern [1, 3]. As vancomycin has relatively clean safety profile and durability against the development of resistance, it has been considered to be the optimal option for the treatment of invasive MRSA infections [4]. However, the emergence of vancomycin- hetero-resistant *S. aureus* (hVRSA), vancomycin-intermediate *S. aureus* (VISA) and vancomycin-resistant *S. aureus* (VRSA) limits the use of vancomycin for the treatment of serious MRSA infections [5–7]. In addition, the drawbacks of vancomycin, including poor tissue penetration, serious nephrotoxicity and neutropenia and unpredictable synergy with other antimicrobial agents, also limit the selection of the alternatives for the treatment of MRSA infections. Fusidic acid (FA), is an effective agent for the treatment of SSTIs, acute osteomyelitis, chronic osteomyelitis, vertebral infection, septic arthritis, and prosthetic and other device related infections caused by MRSA and methicillin-susceptible *S. aureus* (MSSA) [8–10]. FA dose of 250-500 mg every 8–12 h was recommended for the treatment of MRSA infections [8]. However, recent study reported that front-loaded dosing of ≥1200 mg every 12 h (q12h) has better activity against MRSA infections than non-front-loaded dosing (600 mg q12 h) [11]. Although FA has not been approved for use in the USA by the US FDA, this drug has been widely used in Europe, Canada, Australia and some Asian countries for decades [8]. FA is a valuable alternative to vancomycin for the treatment of MRSA infections. In the 1990s, the prevalence of resistance to FA in *S. aureus* remained low. But there is a concern that there is a significant trend towards increased FA resistance among *S. aureus* with increased duration of use. In a surveillance report on FA resistance in 13 European countries, the prevalence of resistance to FA among *S. aureus* isolates was 10.7 %, with the highest resistance rates of 62.4 % among the isolates from Greece [12]. Although many Asian countries have reported FA resistance among *S. aureus* isolates, these data in China are limited. The aim of the present study is to investigate the dissemination of fusidic acid resistance among *S. aureus* clinical isolates from a tertiary hospital between 2012 and 2013 in Wenzhou, east China.

## Methods

### Collection of *S. aureus* clinical isolates

From Jan. 2012 to Sep. 2013, a total of 392 non-duplicate *S. aureus* isolates (single isolate per patient) from the various specimens of patients at the first Affiliated Hospital of Wenzhou Medical University locating in Wenzhou, east China, were selected for investigating the prevalence of FA resistance. The numbers of *S. aureus* isolates included in 2012 and 2013 were 211 and 181. Isolates were identified as *S. aureus* by using Gram’s stain, positive catalase and coagulase test results, and Vitek microbiology analysis (BioMérieux, Marcy l’Etoile, France). *S. aureus* ATCC25923 was used as a control strain for the identification of bacteria. Because the present study focused on bacteria, the Ethics Committee of the first Affiliated Hospital of Wenzhou Medical University exempted this investigation from review. Informed consents were required from patients for sample collection.

### Antimicrobial susceptibility testing

*S. aureus* susceptibilities to penicillin (10 μg), erythromycin (15 μg), clindamycin (2 μg), rifampicin (5 μg), tetracycline(30 μg), linezolid (30 μg), quinupristin/dalfopristin (15 μg), trimethoprim/sulfamethoxazole (1.25/23.75 μg), gentamicin (10 μg), ciprofloxacin (5 μg), chloramphenicol (30 μg), fusidic acid (5 μg) and nitrofurantoin (300 μg) were determined using the disk diffusion test recommended by the Clinical and Laboratory Standards Institute (CLSI) [13]. Every assay was replicated for three times. The mean value was applied for the assay. All disks were obtained from Oxoid Ltd. Vancomycin MICs for *S. aureus* isolates were determined by the agar dilution method in accordance with the CLSI guidelines [14]. Interpretive standards of antimicrobial susceptibility testing for *S. aureus* isolates tested was in accordance with the guidelines provided by CLSI [13]. Initial screening of FA resistance was performed by disk diffusion method with 5 μg FA containing disks. The isolates with an inhibition zone ≤17 mm in diameter were further determined for FA MIC values by an agar dilution method. The interpretive criterion of FA susceptibility is in accordance with the European Committee for Antimicrobial Susceptibility Testing (EUCAST)/ criteria (susceptible, MIC < 2 μg/ml; resistant, MIC ≥ 2 μg/ml). *S. aureus* ATCC 25923 and *Escherichia coli* ATCC25922 were used as reference strains for antimicrobial susceptibility testing.

### DNA extraction

*S. aureus* isolates with FA resistance were cultured on blood agar overnight at 35 °C. Then, three to four bacterial colonies were suspended in 150 μl sterile distilled water with lysostaphin (1 mg/mL) (Sangon, Shanghai, China) and incubated at 37 °C for an hour. Finally, DNA was extracted following the instructions of the Genomic DNA Extraction kit (Sangon, Shanghai, China), stored at −20 °C and prepared for PCR assays.

### MRSA identification

A simplex PCR was used for the detection of *mecA* using MRSA N315 as positive control strain. The isolates positive for *mecA* were identified as MRSA. And cefoxitin disk diffusion test was also used for the detection of MRSA in accordance with the guideline provided by the Clinical and Laboratory Standards Institute (CLSI) [13].

### Detection of FA resistance determinants by PCR

Detection of *fusA* mutations and acquired FA resistance determinants including *fusB* and *fusC* was detected by PCR assays with primers and reaction conditions described previously [15]. DNA sequencing was used for the identification of genotypes of acquired FA resistance determinants. The *fusA* mutations were determined by sequencing entire *fusA* gene.

### SCC*mec* typing

SCC*mec* typing of MRSA isolates was performed using a battery of multiplex PCRs as described previously [16]. The MRSA isolates with unanticipated fragments or lacking fragments by multiplex PCRs were defined as non-typeable (NT). MRSA NCTC 10442 (SCC*mec*I), MRSA N315 (SCC*mec* II), MRSA 85/2082 (SCC*mec* III), MRSA JCSC 4744 (SCC*mec* IV) and MRSA WZ153 (SCC*mec* V) were used as control strains for SCC*mec* typing.

### *spa* typing

The *spa* variable repeat region from each *S. aureus* isolate was amplified using simplex PCR oligonucleotide primers as previously described [17, 18]. Following their purification and sequencing, *spa* types were assigned using the *spa* database website (http://spaserver.ridom.de).

### Multilocus sequence typing (MLST)

MLST typing of *S. aureus* isolates was performed using amplification of internal fragments of the seven housekeeping genes(*arcc*, *aroe*, *glpf*, *gmk, pta*, *tpi* and *yqil*) of *S. aureus* as described previously [19]. Following purification and sequencing of these genes, the sequences were compared with the existing sequences available on the MLST website for *S. aureus* (http://*saureus*.mlst.net), and STs were assigned according to the allelic profiles.

## Results and discussion

### Prevalence of FA resistance among *S. aureus* isolates

Among 392 *S. aureus* isolates tested including 181 MRSA isolates, 56 (14.3 %) with FA MIC values ranging from 2 to ≥128 μg/ml, including 49 (87.5 %, 49/56) MRSA and 7 (12.5 %, 7/56) MSSA, were resistant to FA. The proportions of FA resistance among MRSA and MSSA isolates were 27.1 % (49/181) and 3.3 % (7/211). The sources for the isolation of the 56 FA-resistant isolates included sputum (42, 75 %), pus (10, 17.9 %), catheter (1, 1.8 %), blood (1, 1.8 %) and exudates (2, 3.6 %). Compared with previous reports from China with FA resistance rates of 3.5 % (4/116) from Shanghai and 2.2 % (4/186) from Chinese children with SSTIs [20, 21], the prevalence of FA resistance among *S. aureus* clinical isolates in the present study was very high. The resistance rates to FA among *S. aureus* isolates are different from country to country. The resistance rates of *S. aureus* isolates to FA were very low in the USA (0.3 %) and relatively high in Canada (7.0 %) and Australia (7.0 %) [22]. In an updated surveillance report on FA resistance in 13 European countries, Israel, Italy, Poland, Spain and Sweden had low rates (1.4–3.1 %), while Greece (62.4 %) and Ireland (19.9 %) had the highest resistance rates [12]. In most Asian countries, FA resistance rates are relatively low (<10 %) [8]. FA resistance was observed more frequently among MSSA isolates (0.6 %) than among MRSA isolates from the United States [22]. On the contrary, the prevalence of FA resistance among MRSA isolates was significantly higher than that among MSSA isolates in the present study. There is a significant trend towards increased FA resistance among *S. aureus* since FA has been used widely. In Kuwait, the rate of FA resistance increased rapidly from 22 % in 1994 to 92 % in 2004 [23]. There was a trend of rapidly increased FA resistance among *S. aureus* and MRSA isolates from 5.2 % and 8.9 % in 2012 to 24.9 % and 45.1 % in 2013 in this investigation. The MIC values of FA for 56 FA-resistant isolates were as follows: ≥128 μg/ml, 43; 16 μg/ml, 2; 8 μg/ml, 1; 4 μg/ml, 7; and 2 μg/ml, 3. In 2012, only 5 *S. aureus* isolates (45.5 %, 5/11) with FA MIC value of ≥128 μg/ml were identified. Surprisingly, the FA MIC values for 38 (84.4 %) of 45 FA-resistant isolates were ≥128 μg/ml in 2013. The resistance rates of FA-resistant *S. aureus,* MRSA and MSSA isolates to antimicrobials tested were showed in Table 1. All 56 FA-resistant isolates were susceptible to vancomycin, quinupristin-dalfopristin, linezolid, and nitrofurantoin. The antimicrobials with the resistance rates of more than 50 % among FA-resistant *S. aureus* isolates were penicillin (100 %), erythromycin (92.9 %), clindamycin (89.3 %), ciprofloxacin (82.1 %), and tetracycline (75.0 %), respectively. The resistance rates of the 56 isolates to gentamycin, rifampin, trimethoprim/sulfamethoxazole and chloramphenicol were relatively low. In contrast to the report from Taiwan among which the resistance rate to rimethoprim/sulfamethoxazole among FA-resistant MRSA isolates was high to 97 % [24], only 4.9 % (4/81) of FA-resistant MRSA isolates were resistant to this antimicrobial.

**Table 1:** Antimicrobial resistance profiles of FA-resistant *S.aureus*, MRSA and MSSA isolates

	*S. aureus* (*n* = 56)	MRSA (*n* = 49)	MSSA (*n* = 7)
	R^a^(%)	R(%)	R(%)
Tetracycline	75	81.	28.6
Gentamicin	28.6	30.6	14.3
Penicillin	100	100	100
Clindamycin	89.3	91.8	71.4
Erythromycin	92.9	95.9	71.4
Ciprofloxacin	82.1	87.8	42.9
Linezolid	0	0	0
Rifampicin	10.7	10.2	14.3
Trimethoprim/sulfametho-xazole	3.6	4.1	0
Nitrofurantoin	0	0	0
Chloramphenicol	5.4	6.1	0
Quinupristin/dalfopristin	0	0	0
Vancomycin	0	0	0

a: R, resistance

### Prevalence of FA resistance determinants

Two major FA resistance mechanisms have been reported in *S. aureus*, including the alteration of the drug target site, which is due to mutations in *fusA* encoding elongation factor G (EF-G) or rplF encoding ribosome protein L6), and the protection of the drug target site by FusB family proteins including FusB, FusC, and FusD [8]. In staphylococci, high-level FA resistance is usually associated with mutations in *fusA* encoding EF-G, while low-level resistance is generally caused by the horizontally transferable genes including *fusB*, *fusC* and *fusD* [25]. The spontaneous mutations in *fusA* lead to modification of the drug target and reduced susceptibility [26]. The *fusB*-type resistance involves the recruitment of an exogenous resistance determinant whose product binds EF-G and protects it from FA [27]. In this study, the results from PCR and DNA sequencing demonstrated that acquired resistance gene, *fusB* was present in 73.2 % (41/56) of FA-resistant isolates tested. However, *fusC* and *fusA* mutations were not found in any of tested isolates. The previous investigation from China found that all 4 clinical isolates with FA resistance were positive for *fusB* and negative for *fusC* and *fusA* mutations [20]. These data indicated that *fusB* is the predominant determinant responsible for FA resistance among *S. aureus* in China. In Netherlands, *fusB* was also found to be predominant FA resistance determinant which was detected in about 90 % of FA-resistant *S. aureus* isolates [28]. On the contrary, a report from northern Taiwan found that 84 % of FA-resistant MRSA isolates had *fusA* mutations and another report from a Taiwanese hospital found that *fusC* was the most common FA resistance determinant [15, 24]. *S. aureus* isolates from Australia with FA MIC values ranging from 2 to 32 μg/ml were predominantly *fusC* positive [22]. In U.S. and European collections, *fusC* was also more prevalent than *fusB* in FA-resistant *S. aureus* isolates [12, 22]. In contrast to previous report from Taiwan among which the distribution of FA resistance determinants including *fusA* mutations, *fusB* and *fusC* was quite different between MRSA and MSSA groups and no MRSA isolates carried *fusB* [15].

### Molecular characteristics of FA-resistant *S. aureus* isolates

The molecular characteristics of FA-resistant *S. aureus* isolates were listed in Table 2. A total of 9 STs were identified among the 56 FA-resistant *S. aureus* isolates, among which ST5 accounting for 66.1 % (37/56) was the most common ST, followed by ST630 (12.5 %, 7/56). Only 3 MRSA isolates belonged to ST239 which is the predominant ST in China. The STs for two isolates were not identified. The antimicrobial resistance profiles of ST5 and ST239 FA-resistant MRSA isolates were showed in Table 3. The clonal complex 5 (CC5) was the predominant CC, accounting for the majority (92.9 %, 52/56) of the isolates tested. Twelve *spa* types were identified among the 56 FA-resistant *S. aureus* isolates, among which *t*2460 (67.9 %, 38/56) was the most prevalent *spa* type. The *spa* types for 5 isolates were not identified. *t*2460 was identified among 43.9 % of MRSA isolates from 3 intensive care units at a Korean tertiary hospital in 2007–2008 [29]. Among the 49 MRSA isolates, 40, 7 and 2 harbored SCC *mec* types II, III and V, respectively.

**Table 2:** Molecular characteristic of FA-resistant *S.aureus* isolates

CC^a^ (No.)	STs^b^ (No.)	*spa* types (No.)	MRSA (No.)	MSSA (No.)	SCC*mec* (No.)	*fusB* (No.)	*fusC* (No.)	FA MICs (No.)
5(52)	ST5(37)	t2460(37)	37	0	II(36)III(1)	32	0	≥128(36);4(1)
	ST630(7)	t4549(3)	2	1	III(1)V(1)	1	0	2(1);4(1);16(1)
		t377(2)	0	2		1	0	4(1);16(1)
		t257(1)	0	1		0	0	4(1)
		t2196(1)	0	1		1	0	2(1)
	ST239(3)	t030(3)	2	1	III(2)	2	0	≥128(2); 4(1)
	ST25(2)	t078(2)	2	0	II(2)	1	0	≥128(2)
	ST15(1)	t2460	1	0	II	0	0	4(1)
	ST72(1)	t664	1	0	III	0	0	≥128(1)
	ST2831(1)	t030	1	0	III	1	0	2(1)
7(1)	ST7(1)	t091(1)	0	1		0	0	≥128(1)
59(1)	ST59(1)	t437	1	0	V	1	0	8(1)
Singleton(2)	NT(2)	t11497(1)	1	0	II	0	0	4(1)
		t5137(1)	1	0	III	1	0	≥128(1)

a: CC, clone complex; b: ST, sequence type

**Table 3:** Antimicrobial resistance profiles of FA-resistant ST5 and ST239 MRSA isolates

	ST5 MRSA isolates (*n* = 37)	ST239 MRSA isolates (*n* = 3)
	R^a^(%)	R(%)
Tetracycline	94.6	33.3
Gentamicin	70.3	100
Penicillin	100	100
Clindamycin	100	100
Erythromycin	100	100
Ciprofloxacin	97.3	100
Linezolid	0	0
Rifampicin	5.4	100
Trimethoprim/sulfametho-xazole	3.7	0
Nitrofurantoin	0	0
Chloramphenicol	5.4	0
Quinupristin/dalfopristin	0	0
Vancomycin	0	0

a: R, resistance

In the present study, ST5-MRSA- II-*t*2460 was predominant clone, accounting for 75.5 % (37/49) of FA-resistant MRSA isolates and 66.1 % (37/56) of FA-resistant *S. aureus* isolates. ST5-MRSA- II is usually associated with *t*002 [30]. ST5-MRSA- II-*t*002 was one of two major clones in China [30]. However, ST5-MRSA- II was linked to *t*2460 in this study. This fairly unusual clone was only found among 6 MRSA isolates from Shanghai in China [30]. FA MIC values for 36 of 37 ST5-MRSA- II-*t*2460 isolates were ≥128 μg/ml. ST5-MRSA- II-*t*2460 clone first emerged in 2012. However, this newly emerging clone with FA resistance increased dramatically in 2013, accounting for 77.8 % (35/45) of FA-resistant isolates. The origin of 35 isolates belonged to ST5-MRSA- II-*t*2460 in 2013 was confined to only 4 wards including neurosurgery ward (28 isolates), orthopaedic ward (three isolates), intensive care unit (three isolates) and hematology ward (one isolate), indicating that outbreak of infections caused by this newly clone has occurred in our hospital, mainly in neurosurgery ward. Clonal spread is responsible for the increase in FA resistance in UK, France and other European countries [31, 32]. CC80-MRSA-IV clone carrying *fusB* has made a major contribution to the prevalence of FA resistance among MRSA isolates in Europe [25]. The highest prevalence of FA resistance in 2013 in this investigation was associated with the spread of ST5-MRSA- II-*t*2460 clone with high-level FA resistance. Among 7 FA-resistant MSSA isolates, 5 belonged to ST630-MSSA.

## Conclusions

In conclusion, increased FA resistance among *S. aureus* isolates was found in China. *fusB* was predominant FA resistance determinant. The spread of CC5 clone, especially novel ST5-MRSA- II-*t*2460 clone with high-level resistance to FA, was responsible for the increase of FA resistance.
